# The autophagic response to polystyrene nanoparticles is mediated by transcription factor EB and depends on surface charge

**DOI:** 10.1186/s12951-015-0149-6

**Published:** 2015-11-23

**Authors:** Wensi Song, Lauren Popp, Justin Yang, Ayushi Kumar, Varun Shenoy Gangoli, Laura Segatori

**Affiliations:** Department of Chemical and Biomolecular Engineering, Rice University, Houston, TX 77005 USA; Department of Biochemistry and Cell Biology, Rice University, Houston, TX 77005 USA; Department of Bioengineering, Rice University, Houston, TX 77005 USA

**Keywords:** Autophagy, Lipopigment, Lysosome, Nanoparticle, Transcription factor EB (TFEB)

## Abstract

**Background:**

A number of engineered nanoparticles induce autophagy, the main catabolic pathway that regulates bulk degradation of cytoplasmic material by the lysosomes. Depending on the specific physico-chemical properties of the nanomaterial, however, nanoparticle-induced autophagy may have different effects on cell physiology, ranging from enhanced autophagic degradation to blockage of autophagic flux. To investigate the molecular mechanisms underlying the impact of nanoparticle charge on the nature of the autophagic response, we tested polystyrene nanoparticles (50 nm) with neutral, anionic, and cationic surface charges.

**Results:**

We found all polystyrene nanoparticles investigated in this study to activate autophagy. We showed that internalization of polystyrene nanoparticles results in activation of the transcription factor EB, a master regulator of autophagy and lysosome biogenesis. Autophagic clearance, however, was observed to depend specifically on the charge of the nanoparticles. Particularly, we found that the autophagic response to polystyrene nanoparticles presenting a neutral or anionic surface involves enhanced clearance of autophagic cargo. Cell exposure to polystyrene nanoparticles presenting a cationic surface, on the other hand, results in transcriptional upregulation of the pathway, but also causes lysosomal dysfunction, ultimately resulting in blockage of autophagic flux.

**Conclusions:**

This study furthers our understanding of the molecular mechanisms that regulate the autophagic response to nanoparticles, thus contributing essential design criteria for engineering benign nanomaterials.

**Electronic supplementary material:**

The online version of this article (doi:10.1186/s12951-015-0149-6) contains supplementary material, which is available to authorized users.

## Background

Engineered nanoparticles are widely explored for a variety of biomedical applications, including drug delivery [1–3], in vitro and in vivo diagnostics [4, 5], and production of biocompatible materials [6, 7]. Because of their unique physical and chemical properties, nanoparticles interact with biological components and systems, which also operate at the nanoscale. As a result, nanoparticles induce a variety of biological responses [8–16] including autophagy [17–21], the main catabolic pathway that mediates degradation of aggregated proteins, damaged organelles, and pathogens by lysosomes [22]. Markers of autophagy have been detected upon cellular uptake of a variety of engineered nanoparticles, including metal oxide nanoparticles [23, 24], quantum dots [19, 25], fullerenes [21, 26], gold nanoparticles [18, 27], silver nanoparticles [28], and polymeric nanoparticles [29]. While autophagy is generally considered a pro-survival pathway [30–32], activation of autophagy has also been observed in association with cell death, suggesting that autophagy may play a role in the mechanism of nanoparticle-induced toxicity [33]. However, the molecular mechanisms that govern the autophagic response to nanoparticle internalization remain unclear.

Autophagic clearance is mediated by compartmentalization of cytoplasmic material into double-membrane vesicles called autophagosomes [34]. Fusion of autophagosomes with lysosomes results in the formation of autophagolysosomes where degradation occurs. As a result, autophagic clearance depends upon the coordinated regulation of autophagosome and lysosome biogenesis and function. The transcription factor EB (TFEB) is a master regulator of the lysosome-autophagy system, controlling expression of the CLEAR (coordinated lysosomal expression and regulation) gene network [35]. Activation of TFEB increases the numbers of lysosomes [35] and autophagosomes, which are needed for degradation of autophagic cargo [36]. Activation of TFEB has been observed upon internalization of synthetic nanoparticles [37] and autophagic clearance induced by ceria nanoparticles was found to specifically depend on TFEB activation [38].

Transcriptional activation of autophagy, however, is not always followed by an increase in autophagic clearance [39–43]. Nanoparticle uptake may result in impairment of downstream steps of the autophagy pathway, such as lysosomal function [27]. We hypothesize that nanoparticle-induced impairment of lysosomal function may affect lysosome-autophagosome fusion, possibly leading to blockage of autophagic flux and cytotoxicity.

A number of endocytosed nanoparticles are found to accumulate in lysosomes [44–47]. Cationic nanoparticles have been reported to induce autophagy, but also to disrupt lysosomes [12, 48, 49]. A “proton sponge” effect has been proposed as a potential mechanism for the observed lysosomal disruption and membrane permeabilization. Specifically, it was suggested that the high proton buffering capacity of the amino groups on the nanoparticle surface interferes with acidification of lysosomes, impairs the proton pump activity, and ultimately induces lysosomal membrane permeabilization [12].

We hypothesized that the surface charge of nanoparticles plays an important role in determining the nature of the autophagic response that is induced upon nanoparticle uptake. This hypothesis was investigated by testing the autophagic response activated upon cell exposure to non-functionalized polystyrene nanoparticles presenting neutral surfaces (PS), carboxyl-functionalized nanoparticles presenting anionic surfaces (PS-COOH), and amino-functionalized nanoparticles presenting cationic surfaces (PS-NH_2_). This study elucidates the cellular and molecular mechanisms that determine whether nanoparticle-mediated autophagy activation results in effective autophagic clearance or blockage of autophagic flux, thereby mapping the nanoparticle surface charge to biocompatible and bioadverse outcomes of nanoparticle-induce autophagy activation.

## Results

### Characterization of polystyrene nanoparticles

Zeta potential measurements were conducted to verify the surface charge of polystyrene nanoparticles (50 nm) functionalized with neutral (PS), anionic (PS-COOH) and cationic (PS-NH_2_) groups in water, PBS, and cell culture medium (Table 1). All the polystyrene NPs present negatively charged zeta potentials in cell culture medium due to the formation of a negatively charged protein corona, as previously reported [12].

**Table 1:** Zeta potential and hydrodynamic diameter measurements of polystyrene nanoparticles in DI water, PBS, and cell culture medium

	Zeta potential (mV)			Hydrodynamic diameter (nm)		
	DI water	PBS	Cell culture medium	DI water	PBS	Cell culture medium
PS	−8.0 ± 2.0	−16.7 ± 11.0	−13.9 ± 0.6	50.1 ± 1.0	49.4 ± 3.6	88.9 ± 1.8
PS-COOH	−21.3 ± 2.2	−17.1 ± 7.7	−8.2 ± 2.6	60.3 ± 5.0	53.3 ± 14.5	53.5 ± 14.3
PS-NH_2_	+20.4 ± 1.3	+13.7 ± 3.3	−13.9 ± 0.3	74.8 ± 16.2	54.0 ± 12.4^a^	369.1 ± 29.3

^a^A bimodal distribution of PS-NH2 in PBS indicates primary particles (54.0 ± 12.4 nm) and aggregates with an average hydrodynamic diameter of 340.1 ± 171.3 nm

Nanoparticle concentrations are reported in units of µg/mL and were calculated assuming the density of the polystyrene nanoparticles in cell culture medium to approximately 1 g/cm^3^.

### Polystyrene nanoparticles activate TFEB in HeLa/TFEB cells

**Fig. 1:**
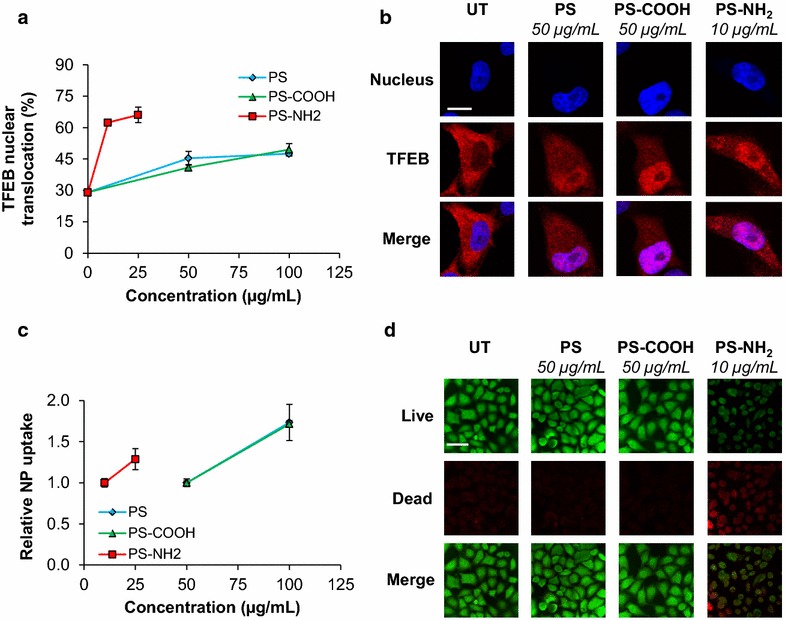
Polystyrene nanoparticles promote TFEB activation in HeLa/TFEB cells. **a** Average fraction of TFEB that localizes in the nucleus of HeLa/TFEB cells untreated and treated with PS (50 and 100 µg/mL), PS-COOH (50 and 100 µg/mL), and PS-NH_2_ (10 and 25 µg/mL) for 24 h. Representative fields (~30) each containing approximately 50 cells were analyzed and data reported as mean ± SD (n = 3; p < 0.01). **b** Representative confocal microscopy images of TFEB subcellular localization in HeLa/TFEB cells treated with nanoparticles as described in **a**. UT, untreated. The *scale bar* is 10 μm. **c** Cellular uptake of polystyrene nanoparticles in HeLa/TFEB cells treated with nanoparticles as described in **a**. Data are reported as mean ± SD (n = 3; p < 0.05). **d** Confocal microscopy analyses of calcein (*green*) and EthD-1 binding (*red*) in HeLa/TFEB cells treated with PS (50 µg/mL), PS-COOH (50 µg/mL), and PS-NH_2_ (10 µg/mL) for 24 h. *UT* untreated. The *scale bar* is 40 μm

To investigate the molecular mechanisms involved in the autophagic response to nanoparticles of different surface charge, we first analyzed the transcriptional regulatory network that controls autophagy activation by monitoring TFEB intracellular localization in cells treated with PS, PS-COOH, and PS-NH_2_. TFEB localizes predominantly in the cytoplasm of resting cells and translocates into the nucleus upon activation [35]. We used HeLa cells stably transfected for the expression of TFEB-3xFLAG (HeLa/TFEB cells) because they provide an in vitro model system of TFEB activation [35]. HeLa/TFEB cells were treated with nanoparticles at final medium concentrations ranging from 10 to 100 µg/mL and TFEB intracellular localization was evaluated by confocal microscopy using Hoechst nuclear stain and an anti-FLAG antibody (Fig. 1a, b).

TFEB was found to localize predominantly in the cytoplasm of untreated HeLa/TFEB cells, as expected [35]. Specifically, the average fraction of TFEB that localizes in the nucleus of untreated cells was 29.1 ± 1.7 % (Fig. 1a). A significant increase in the fraction of TFEB that localizes in the nucleus was observed upon treatment with PS or PS-COOH at medium concentrations of 50 µg/mL and higher. Specifically, the average fraction of TFEB that localizes in the nucleus was found to increase to over 40 % after 24 h of cell treatment with PS (50 µg/mL: 45.4 ± 3.3 %; 100 µg/mL: 47.5 ± 1.0 %, Fig. 1a; p < 0.01) or PS-COOH (50 µg/mL: 40.9 ± 1.4 %; 100 µg/mL: 49.5 ± 2.8 %, Fig. 1a; p < 0.01). Interestingly, the extent of TFEB activation was found not to reach a plateau upon cell treatment with concentrations of PS and PS-COOH higher than 100 µg/mL. A dramatic increase in TFEB nuclear localization was observed in cells treated with lower concentrations of PS-NH_2_ (10 and 25 µg/mL) compared to PS and PS-COOH. After 24 h of treatment with PS-NH_2_, the average fraction of TFEB that localizes in the nucleus was found to increase to over 60 % (10 µg/mL: 62.4 ± 1.0 %; 25 µg/mL: 66.0 ± 3.7 %, Fig. 1a; p < 0.01). Cell treatment with higher medium concentrations of PS-NH_2_ (50–100 µg/mL) resulted in considerable cytotoxicity and cell death, precluding accurate evaluation of TFEB nuclear localization. Representative images are reported in Fig. 1b.

These results indicate that cationic polystyrene nanoparticles induce TFEB activation in HeLa/TFEB cells to a higher extent than neutral and anionic nanoparticles and that the minimum concentration of cationic polystyrene nanoparticles needed to induce activation of TFEB is lower than that of neutral and anionic nanoparticles.

To investigate the correlation between activation of TFEB and uptake of polystyrene nanoparticles of different surface charge, we measured the extent of nanoparticle internalization in HeLa/TFEB cells under conditions observed to activate TFEB. Cellular uptake of fluorescently labeled polystyrene nanoparticles (PS, PS-COOH, and PS-NH_2_) was observed to follow a concentration-dependent behavior under the conditions used in this study (Fig. 1c). Interestingly, the extent of TFEB activation was not found to vary dramatically above a minimum concentration that depends on the nanoparticle surface charge. These results suggest that TFEB activation may function as a switch-like response that is activated upon uptake of a critical nanoparticle concentration.

Polystyrene nanoparticle-induced toxicity was analyzed under conditions that result in TFEB activation in HeLa/TFEB cells. As expected, we found that cell treatment with PS or PS-COOH (50 µg/mL; 24 h) does not cause cytotoxicity in HeLa/TFEB cells (Fig. 1d). Cell treatment with PS-NH_2_ (10 µg/mL; 24 h), however, results in considerable cytotoxicity (Fig. 1d), possibly due to elevation of lysosomal pH and impairment of lysosomal integrity [10, 12, 48]. These results suggest that the dramatic increase in TFEB activation observed in cells treated with low concentrations of PS-NH_2_ compared to PS or PS-COOH may be due to lysosomal stress [50] caused by nanoparticle treatment under these conditions.

In summary, these studies indicate that cell treatment with neutral and anionic polystyrene nanoparticles (50 µg/mL) does not induce cytotoxicity and results in activation of TFEB in HeLa/TFEB cells. However, treatment of the same cell line with cationic polystyrene nanoparticles at significantly lower concentrations (10–25 µg/mL) induces a higher extent of TFEB activation and cytotoxicity compared to neutral and anionic nanoparticles. Higher concentrations of cationic nanoparticles (>25 µg/mL) were found to cause excessive cytotoxicity and cell death.

### Polystyrene nanoparticles activate TFEB in PC12 cells

**Fig. 2:**
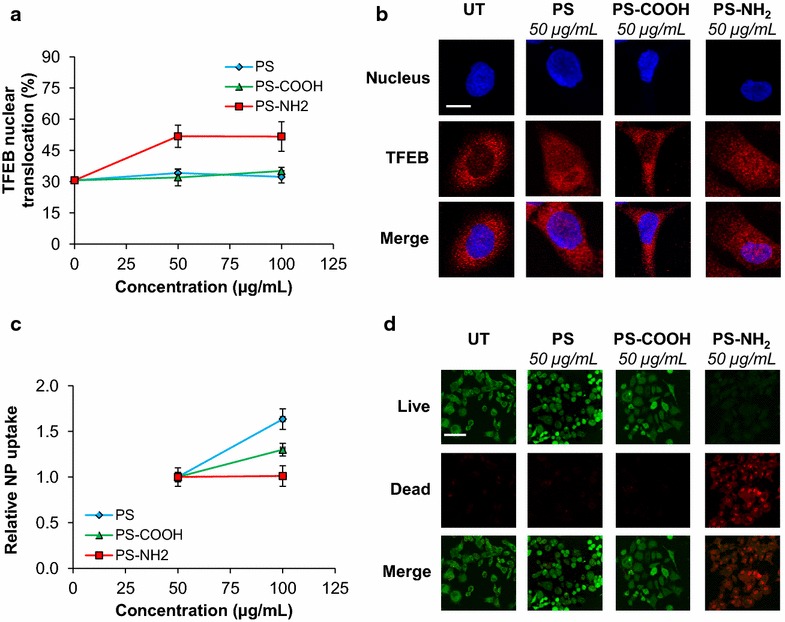
PS-NH_2_ promote TFEB activation in PC12 cells. **a** Average fraction of TFEB that localizes in the nucleus of PC12 cells treated with PS, PS-COOH, and PS-NH_2_ (50 and 100 µg/mL) for 24 h. Representative fields (~30) containing approximately 50 cells were analyzed and data reported as mean ± SD (n = 3; p < 0.01). **b** Representative confocal microscopy images of TFEB subcellular localization in HeLa/TFEB cells treated with nanoparticles as described in **a**. UT, untreated. The *scale bar* is 10 μm. **c** Cellular uptake of polystyrene nanoparticles in PC12 cells treated with nanoparticles as described in **a**. Data are reported as mean ± SD (n = 3; p < 0.05). **d** Confocal microscopy analyses of calcein (*green*) and EthD-1 binding (*red*) in HeLa/TFEB cells treated with PS (50 µg/mL), PS-COOH (50 µg/mL), and PS-NH_2_ (50 µg/mL) for 24 h. *UT* untreated. The *scale bar* is 40 μm

To further investigate how polystyrene nanoparticle surface charge affects TFEB activation under conditions that do not induce significant cellular stress, we tested the impact of polystyrene nanoparticles with neutral, anionic, and cationic surface charge on TFEB intracellular localization in PC12 cells. PC12 cells were selected for these studies because they are more resistant to many of the cytotoxic effects induced by the uptake of cationic nanoparticles including lysosomal permeabilization, mitochondrial damage, and increased intracellular and mitochondrial calcium levels [12]. PC12 cells were treated with nanoparticles (10–100 µg/mL; 24 h) and TFEB intracellular localization was evaluated and quantified as described above (Fig. 2a). TFEB localizes predominantly in the cytoplasm of untreated PC12 cells, as observed in HeLa/TFEB cells. Specifically, the average fraction of TFEB that localizes in the nucleus of untreated cells was found to be 30.7 ± 0.7 % of the total TFEB (Fig. 2a). TFEB nuclear translocation was found to increase to over 50 % upon cell treatment with PS-NH_2_ (50 µg/mL: 51.8 ± 5.3 %; 100 µg/mL: 51.7 ± 7.1 %), but not in cells treated with PS or PS-COOH at the same concentration (Fig. 2a; p < 0.01). Representative images are reported in Fig. 1b.

Similar to what was observed in experiments conducted using HeLa/TFEB cells (Fig. 1c), analyses of nanoparticle uptake revealed an increase in nanoparticle uptake as a function of nanoparticle concentration in PC12 cells treated with PS and PS-COOH (Fig. 2c). However, the internalization of PS-NH_2_ was found not to increase with nanoparticle concentration, suggesting again that TFEB activation may function as a switch-like response that is activated upon uptake of a critical nanoparticle concentration.

Cytotoxicity analyses in PC12 treated with polystyrene nanoparticles revealed that, similar to HeLa/TFEB cells (Fig. 1), neither PS nor PS-COOH alter cell viability, while treatment with PS-NH_2_ results in considerable toxicity at the concentrations tested (Fig. 2d).

Collectively, these results indicate that cell exposure to polystyrene nanoparticles of different surface charge (neutral, anionic, cationic) results in activation of TFEB. These results also show that cationic polystyrene nanoparticles induce TFEB activation to a higher level than neutral or anionic polystyrene nanoparticles in all cell lines tested, both in terms of the extent of TFEB nuclear translocation and in terms of the minimal concentration of nanoparticle needed to observe TFEB activation. Moreover, the minimum medium concentration of cationic nanoparticles that result in activation of TFEB in PC12 cells, which are more resistant to cationic nanoparticle-induced cytotoxicity [12], was found to be higher than that observed in HeLa/TFEB cells, suggesting a correlation between sensitivity to cationic nanoparticles and TFEB activation.

### Clearance of autophagic cargo upon internalization of polystyrene nanoparticles depends on nanoparticle surface charge

**Fig. 3:**
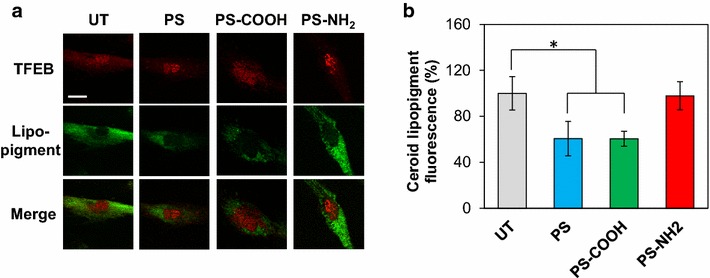
Accumulation of ceroid lipopigment in LINCL fibroblasts treated with polystyrene nanoparticles. **a** Confocal microscopy analyses of TFEB (*red*) and ceroid lipopigment (*green*) in LINCL fibroblasts treated with PS, PS-COOH or PS-NH_2_ (25 µg/mL) for 3 days and evaluated by detecting *green* autofluorescence and binding of anti-TFEB antibody, respectively. *UT* untreated. The *scale bar* is 20 μm. **b** Quantification of ceroid lipopigment fluorescence intensity calculated as described in the “Methods”. Data are reported as mean ± SD (n = 15; *p < 0.01)

To test whether polystyrene nanoparticle-induced TFEB activation parallels autophagic clearance, we monitored the degradation of autophagic cargo. We used fibroblasts derived from a patient with late infantile neuronal ceroid lipofuscinosis (LINCL) which are characterized by accumulation of ceroid lipopigment [51]. Ceroid lipopigment is a lipofuscin-like autofluorescent material [52] normally degraded through autophagy; LINCL cells thus provide a reliable in vitro model system to quantify autophagic clearance. Preliminary studies were conducted to evaluate the minimum medium concentration of polystyrene nanoparticles required to activate TFEB in LINCL fibroblasts, as results obtained with HeLa/TFEB and PC12 cells suggest that the extent of TFEB activation is cell type dependent. Cells were exposed to nanoparticles for 3 days, an incubation time necessary to monitor autophagic clearance [38]. Cell treatment with polystyrene nanoparticles (neutral, anionic and cationic) at 25 µg/mL causes an increase in the fraction of TFEB that localizes into the nucleus, compared to untreated cells (Fig. 3a, red). TFEB activation was observed upon treatment with low concentrations (25 µg/mL) of all three types of polystyrene nanoparticles possibly due to the prolonged time of incubation (3 days) of LINCL fibroblasts compared to HeLa/TFEB cells and PC12 cells (24 h).

Accumulation of ceroid lipopigment was monitored in LINCL fibroblasts treated with polystyrene nanoparticles under conditions found to activate TFEB. Confocal microscopy analyses showed that accumulation of ceroid lipopigment was reduced in cells treated with PS or PS-COOH (Fig. 3a, green). Specifically, the fluorescence intensity of ceroid lipopigment decreased to approximately 60 % of the fluorescence intensity of untreated cells after treatment with PS or PS-COOH (PS: 60.7 ± 15.0 %; PS-COOH: 60.5 ± 6.5 %, Fig. 3b; *p* < 0.01). Treatment with PS-NH_2_ under the same condition, however, was found not to affect the accumulation of ceroid lipopigment (Fig. 3a, green) despite the remarkable effect of PS-NH_2_ on TFEB activation. PS-NH_2_ treatment at higher concentrations resulted in considerable induction of cytotoxicity precluding accurate evaluation of ceroid lipopigment accumulation.

### Cationic polystyrene nanoparticles impair lysosomal integrity and block autophagic flux

Autophagic clearance relies on the coordinated activation of lysosome and autophagosome biogenesis and function [53]. In addition to being transcriptionally upregulated, both branches of the lysosome-autophagy system need to be functionally active to promote autophagic clearance [39–43]. If lysosomal activity is impaired, transcriptional upregulation of autophagy genes and increased formation of autophagosomes are ultimately associated with decreased autophagic clearance [27].

**Fig. 4:**
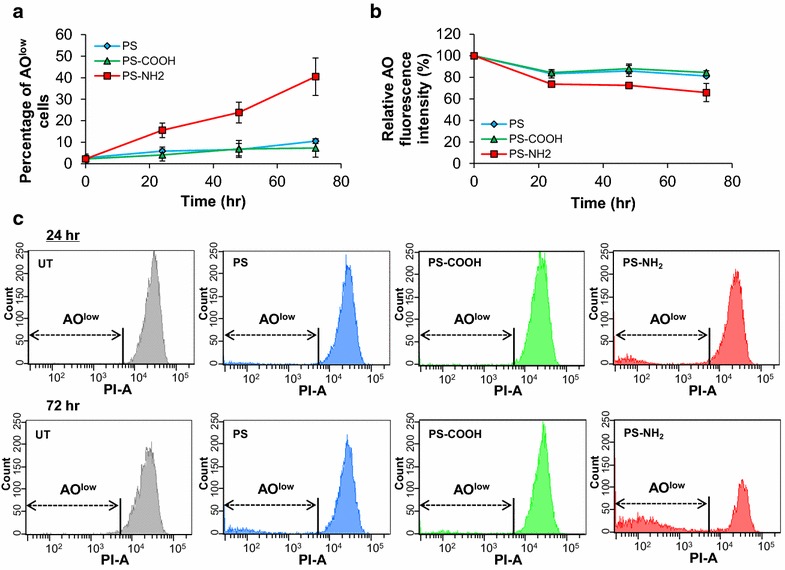
Lysosomal integrity in fibroblasts treated with polystyrene nanoparticles. **a** Percentage of cells with low AO fluorescence (AO_low_). AO fluorescence intensity was measured by flow cytometry every 24 h for up to 72 h in fibroblasts treated with PS, PS-COOH or PS-NH_2_ (25 µg/mL). The results obtained from each replicate were acquired from the analysis of 10,000 cells. The percentage of cells with low AO fluorescence (AO_low_) was calculated by normalizing the cell number with low AO fluorescence by the total cell number. Data are reported as mean ± SD (n = 3; p < 0.05). **b** Average AO fluorescence intensity measured by flow cytometry of cells treated with polystyrene nanoparticles as described in **a**. Data are reported as mean ± SD (n = 3; p < 0.05). **c** Representative histograms of cells treated with different types of polystyrene nanoparticle after 24 and 72 h. *UT* untreated

To investigate the impact of the surface charge of polystyrene nanoparticles on lysosomal function, we assessed lysosomal integrity in fibroblasts treated with PS, PS-COOH, and PS-NH_2_. Fibroblasts were treated with polystyrene nanoparticles (25 µg/mL) and collected every 24 h for up to 72 h. Lysosomal integrity was evaluated using acridine orange (AO), a lysosomotropic probe that accumulates in acidic organelles and exhibits red fluorescence. The red fluorescence of AO dissipates upon lysosomal membrane permeabilization when the dye is released into a more neutral environment. Therefore, a decrease in fluorescence intensity indicates abnormalities in lysosomal pH, or disruption of the lysosomal membrane. The fluorescence intensity of AO in cells treated with polystyrene nanoparticles was measured using flow cytometry, and lysosomal impairment was evaluated by calculating the percentage of cells that present low AO fluorescence as described in the Methods (AO^low^ cells; Fig. 4a). The total AO fluorescence relative to untreated cells was also quantified to evaluate lysosomal integrity in the entire cell population (Fig. 4b). Treatment with PS-NH_2_ but not with PS or PS-COOH was found to increase the percentage of cells presenting low AO fluorescence (Fig. 4a), indicating an increase in the number of cells with lysosomal impairment upon PS-NH_2_ treatment. In addition, a significant decrease in the total AO fluorescence intensity in cells treated with PS-NH_2_ was observed, suggesting an average decrease in lysosomal integrity (Fig. 4b). Representative histograms of cells treated with different types of polystyrene nanoparticle after 24 and 72 h are reported in Fig. 4c. Collectively, these results suggest that cationic polystyrene nanoparticles impair lysosomal integrity.

Because impairment of lysosomal integrity observed in cells treated with cationic nanoparticles may result from an increase in lysosomal pH induced upon accumulation of cationic nanoparticles within lysosomes [10, 54], we asked whether the nanoparticles investigated in this study accumulate in the lysosomes under conditions found to affect lysosomal integrity. Lysosomal accumulation of polystyrene nanoparticles was evaluated by testing the colocalization of polystyrene nanoparticles and LAMP-2, a protein that resides on the membrane of lysosomes, using confocal microscopy [55]. All three types of polystyrene nanoparticles (PS, PS-COOH, and PS-NH_2_) were found to accumulate in lysosomes at each time point investigated (25 µg/mL; 24, 48, and 72 h) (see Additional file 1). Because lysosomal function and integrity depends on the acidic pH of the lysosomal environment [56–58], these results suggest that impairment of lysosomal integrity observed upon uptake of cationic nanoparticles is possibly an effect of the nanoparticle surface charge. These results would also explain why neutral and anionic nanoparticles were found to accumulate in the lysosome at each time point investigated, but were found not to affect lysosomal integrity.

**Fig. 5:**
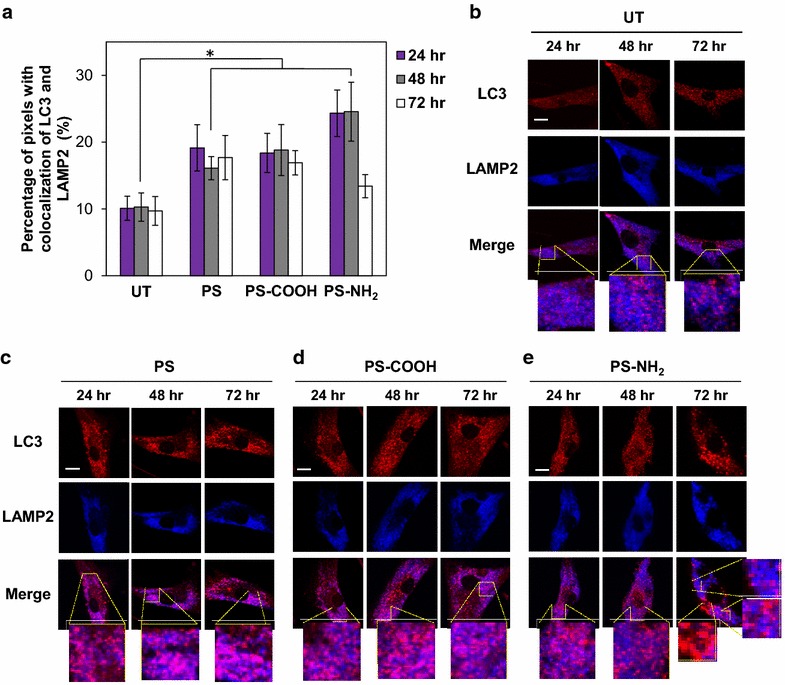
Colocalization of LC3 and LAMP-2 in fibroblasts treated with polystyrene nanoparticles. **a** Quantification of LC3-LAMP-2 colocalization in fibroblasts treated with polystyrene nanoparticles (50 nm; 25 µg/mL) calculated as described in the “Methods”. *UT* untreated. Data are reported as mean ± SD (n = 15; *p < 0.01). **b**–**e** Representative confocal microscopy images of LC3 (*red*) and LAMP-2 (*blue*) in **b** untreated fibroblasts (UT) and in fibroblasts treated with **c** PS (25 µg/mL), **d** PS-COOH (25 µg/mL), **e** PS-NH_2_ (25 µg/mL) after 24, 48, and 72 h evaluated by detecting binding of anti-LC3 antibody and binding of anti-LAMP-2 antibody, respectively. Colocalization of LC3 (*red*) and LAMP-2 (*blue*) is shown in merged images (*purple*). The *scale bar* is 20 μm

Lysosomal membrane permeabilization may compromise lysosome–autophagosome fusion. To test whether polystyrene nanoparticle treatment impairs lysosome–autophagosome fusion, autophagolysosome formation was monitored under conditions that caused a change in lysosomal integrity. We evaluated the colocalization of LC3 and LAMP-2, which are proteins on the membranes of autophagosomes [59], and lysosomes [55], respectively. Fibroblasts were treated with nanoparticles (25 µg/mL) for up to 72 h. Colocalization was evaluated using confocal microscopy and quantified as described in the “Methods” (Fig. 5a). A significant increase in the extent of LC3 and LAMP-2 colocalization was observed in cells treated with PS or PS-COOH compared to untreated cells (untreated: 10.1 ± 1.8 % after 24 h, 10.3 ± 2.1 % after 48 h, 9.7 ± 2.2 % after 72 h. PS: 19.1 ± 3.4 % after 24 h, 16.1 ± 1.7 % after 48 h, 17.7 ± 3.3 % after 72 h. PS-COOH: 18.4 ± 2.9 % after 24 h, 18.8 ± 3.8 % after 48 h, 16.9 ± 1.8 % after 72 h) (Fig. 5a; *p* < 0.01), indicating that neutral and anionic polystyrene nanoparticles promote fusion of autophagosomes and lysosomes and formation of autophagolysosomes. The extent of colocalization of LC3 and LAMP-2 in cells treated with PS-NH_2_ reached 24.3 ± 3.5 % after 24 h of incubation and 24.6 ± 4.4 % after 48 h, but decreased to 13.7 ± 1.7 % after 72 h (Fig. 5a; *p* < 0.01), indicating that formation of autophagolysosomes is ultimately impaired by cationic nanoparticle treatment. Representative images of untreated cells and cells treated with PS, PS-COOH and PS-NH_2_ are reported in Fig. 5b–e.

These results indicate that lysosomal accumulation of nanoparticles presenting neutral and negative surface charges cause induction of the autophagic response through activation of TFEB, which manifests as an increase in lysosome–autophagosome fusion and, ultimately, enhanced clearance of autophagic cargo. Lysosomal accumulation of nanoparticles presenting positive surface charge, however, causes activation of the autophagic response, but also lysosomal dysfunction. As a result, internalization of polystyrene nanoparticles presenting positive surface charge eventually manifests as blockage of autophagic flux and does not result in a change in clearance of autophagic cargo.

## Discussion

In this study, we investigated the molecular mechanisms associated with the autophagic response to polystyrene nanoparticles as a function of nanoparticle surface charge. Cationic nanoparticles were found to induce activation of TFEB, a master regulator of lysosome biogenesis and autophagy, to a higher level than neutral or anionic nanoparticles, both in terms of the extent of TFEB nuclear localization and the minimal concentration of nanoparticle needed to observe an increase in TFEB activation.

Downstream steps of the autophagy pathway, namely lysosomal function, lysosome-autophagosome fusion, and clearance of autophagic cargo were also monitored to establish a correlation between transcriptional activation of autophagy and the nature of the autophagic response induced upon uptake of nanoparticles. Cell treatment with neutral and anionic polystyrene nanoparticles was found to upregulate the autophagy system, enhance autophagic activity, and, ultimately, increase clearance of autophagic substrates. Cell treatment with cationic polystyrene nanoparticles, on the other hand, was found to cause impairment of lysosomal integrity, reduced formation of autophagolysosomes and, ultimately, blockage of autophagic flux. As a result, activation of the autophagic response observed in cells treated with cationic nanoparticles does not parallel an increase in degradation of autophagic substrates.

Nanoparticle aggregation is commonly observed upon cellular internalization, typically due to the high ionic strength conditions of the cytosolic environment [8, 60]; whether nanoparticle aggregation plays a role in the activation of TFEB observed in response to cell treatment with polystyrene nanoparticles remains to be determined.

High levels of both autophagy and cell death are often observed under stress conditions induced by accumulation of positively charged nanoparticles in lysosomes, suggesting that cell death under these conditions is mediated by autophagy. Generally speaking, a number of stressful stimuli that ultimately lead to cell death initially induce activation of autophagy as a pro-survival pathway. This observation has led to the assumption that autophagy contributes to cell death [30]. Cell death by autophagy, however, is expected to result in upregulation of the autophagy system including increase in autophagic flux [61, 62]. Interestingly, cationic nanoparticles induce activation of TFEB that is not paralleled by increase in degradation of autophagic cargo but rather by impairment of lysosomal function, suggesting that cell death under these conditions is not caused by autophagy, but rather by impairment of autophagy.

This study provides a mechanistic understanding of the interaction of polystyrene nanoparticles of different surface charges with the autophagic pathway and contributes to defining the design rules for engineering nanoparticles with desired effect on the autophagy system and nanotherapeutics for the treatment of diseases characterized by inefficient autophagic activity and accumulation of storage material.

## Conclusions

These results demonstrate that cell exposure to neutral and anionic polystyrene nanoparticles results in TFEB activation, enhancement of autophagic activity, and, ultimately, increased degradation of autophagic cargo. Cationic polystyrene nanoparticles induce activation of TFEB, but also cause impairment of lysosomal integrity, reduced formation of autophagolysosomes, and, ultimately, blockage of autophagic flux.

## Methods

### Reagents and cell cultures

Polystyrene nanoparticles were purchased from Magsphere or Phosphorex (see Additional file 2). Bafilomycin was purchased from Cayman Chemical, Acridine Orange was from Invitrogen. Cell culture medium, PBS, and TrypLE Express were from Lonza.

Fibroblasts derived from Late Infantile Neuronal Ceroid Lipofuscinosis (LINCL) patients were from Coriell Cell Repositories (GM16486). HeLa cells stably transfected for the expression of TFEB-3xFLAG were a gift from Dr. Sardiello [35]. Cells were grown at 37 °C in 5 % CO_2_ in Dulbecco’s Modified Eagle Medium supplemented with heat-inactivated fetal bovine serum (20 % FBS for LINCL fibroblasts, 10 % FBS for HeLa cells) and 1 % glutamine Pen-Strep. Monolayers were passaged with TrypLE Express.

PC12 cells were from ATCC. Cells were grown at 37 °C in 5 % CO_2_ in Dulbecco’s Modified Eagle Medium supplemented with 5 % heat-inactivated fetal bovine serum, 10 % horse serum and 1 % glutamine Pen-Strep. Monolayers were passaged with TrypLE Express.

### Characterization of nanoparticles

The surface charge and hydrodynamic diameter of polystyrene nanoparticles was analyzed using a Brookhaven Instruments ZetaPALS system and a Beckman Coulter DelsaMax Pro system.

### Cellular uptake of polystyrene nanoparticles

Nanoparticle uptake was evaluated as previously reported [63]. Briefly, 10^4^ cells were plated in each well of 96-well plates and incubated overnight. Cells were exposed to nanoparticles for 24 h, washed three times with PBS, and lysed with the complete lysis-M buffer containing the protease inhibitor cocktail (Roche). The fluorescence intensity of the cell lysate was quantified using a SpectraMax Gemini plate reader (Molecular Device) (excitation 488 nm, emission 530 nm). The fluorescence intensity of cell lysates was normalized to the absolute fluorescence of each corresponding nanoparticle to calculate the intracellular concentration of polystyrene nanoparticles.

### Immunofluorescence assays

TFEB intracellular localization was evaluated by confocal microscopy using Hoechst nuclear stain (Enzo Life Sciences), and an anti-FLAG antibody (Sigma-Aldrich) or an anti-TFEB antibody (Abcam Inc). The percentage of TFEB nuclear localization was calculated by normalizing the fluorescence intensity of TFEB that localizes in the nucleus by the total fluorescence intensity of TFEB in each cell measured using MATLAB. Average values were calculated over 30 images each containing ~50 cells and collected from at least three independent experiments.

The accumulation of ceroid lipopigment was quantified as previously described [52]. Average values were calculated over 30 images each containing ~5–10 cells and collected from at least three independent experiments.

The colocalization of LC3 (red) and LAMP-2 (blue) was evaluated by confocal microscopy using an anti-LC3 antibody (Novus Biologics) and an anti-LAMP-2 antibody (BioLegend). Colocalization was quantified by calculating the number of pixels with both red and blue signals above a predefined brightness threshold (grey scale >30, range 0–255) and with a red to blue ratio within a predefined range (0.5–2). The percentage of colocalization was calculated by normalizing the number of pixels presenting LC3 and LAMP-2 colocalization to the total number of pixels in each cell over the entire image. Average values were calculated over 30 images each containing ~5–10 cells and collected from at least three independent experiments.

### Cell viability assays

Cell viability assays were performed using the LIVE/DEAD^®^ Viability/Cytotoxicity assay according to the manufacturer’s specifications (Life Technologies). Images were obtained using an Olympus IX81 confocal microscope and co-localized using Fluoview software.

### Lysosomal integrity assays

Lysosomal integrity was analyzed using acridine orange (AO) as previously described [12]. Cells were incubated with nanoparticles for 24 h and exposed to 1 μg/mL AO for 15 min at 37 °C. Samples (10,000 cells) were analyzed by flow cytometry (FACSCanto™ II) using a 488-nm Argon laser and a 670-nm emission filter. The cell population presenting low AO fluorescence was defined by setting the gate boundary corresponding to 2 % of untreated cells presenting low AO fluorescence.

### Statistical analyses

All data is presented as mean ± S.D., and statistical significance was calculated using a two-tailed *t* test.

## Additional files

10.1186/s12951-015-0149-6 Lysosomal accumulation of polystyrene nanoparticles. Confocal microscopy analyses of polystyrene nanoparticles (green) and LAMP-2 (blue) in fibroblasts treated with polystyrene nanoparticles (50 nm; 25 µg/mL), evaluated every 24 h for up to 72 h by detecting the fluorescence intensity of fluorescently labeled polystyrene nanoparticles and binding of anti-LAMP-2 antibody, respectively. Colocalization of polystyrene nanoparticles (green) and LAMP-2 (blue) is shown in merged images. UT, untreated. The scale bar is 20 μm.
10.1186/s12951-015-0149-6 Polystyrene nanoparticle purchasing information.

## Abbreviations

AO: Acridine orange
CLEAR: Coordinated lysosomal expression and regulation
LAMP: Lysosome-associated membrane protein
LC3: Light-chain 3
LINCL: Late infantile neuronal ceroid lipofuscinosis
PS: Polystyrene nanoparticles
PS-NH_2_: Amine-functionalized polystyrene nanoparticles
PS-COOH: Carboxyl-functionalized polystyrene nanoparticles
TFEB: Transcription Factor EB
