# Supplementary material for: The autophagic response to polystyrene nanoparticles is mediated by transcription factor EB and depends on surface charge
Source: J Nanobiotechnology. 2015 Nov 23;13:87. doi: 10.1186/s12951-015-0149-6 (PMC4657241; doi:10.1186/s12951-015-0149-6)
Supplement: Supplementary file 1 — 10.1186/s12951-015-0149-6 Lysosomal accumulation of polystyrene nanoparticles. Confocal microscopy analyses of polystyrene nanoparticles (green) and LAMP-2 (blue) in fibroblasts treated with polystyrene nanoparticles (50 nm; 25 µg/mL), evaluated every 24 h for up to 72 h by detecting the fluorescence intensity of fluorescently labeled polystyrene nanoparticles and binding of anti-LAMP-2 antibody, respectively. Colocalization of polystyrene nanoparticles (green) and LAMP-2 (blue) is shown in merged images. UT, untreated. The scale bar is 20 μm. [file 12951_2015_149_MOESM1_ESM.pdf]

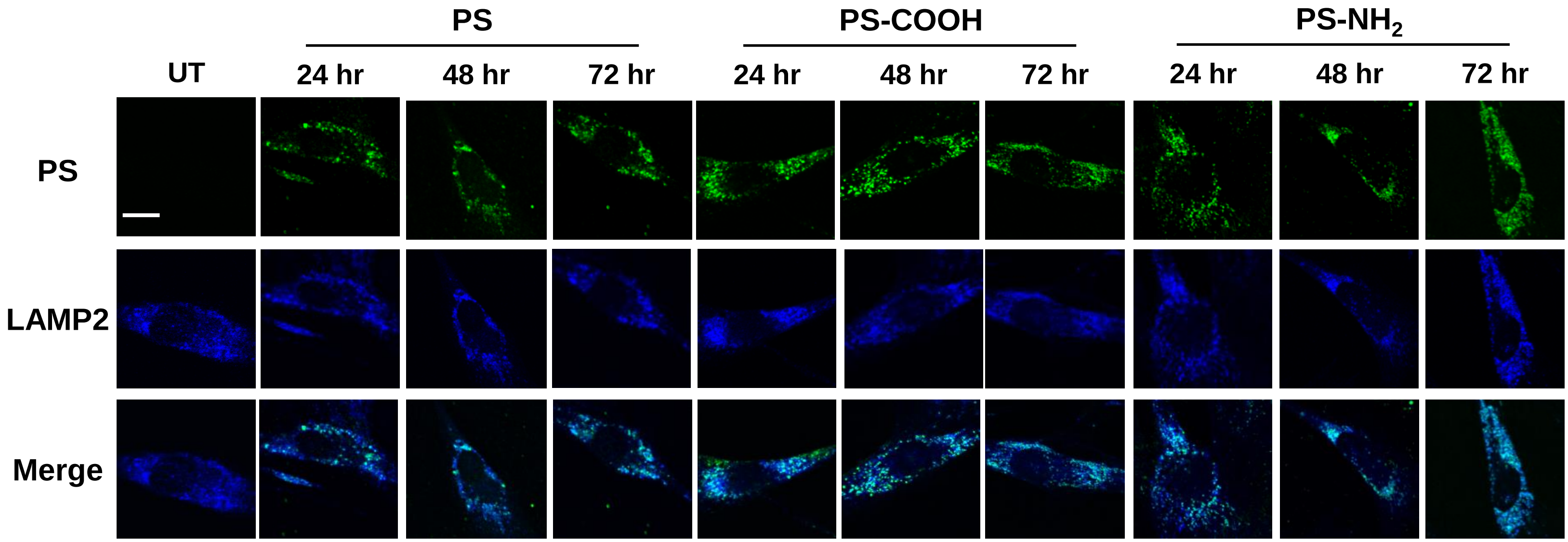


**Song_AdditionalFile1.pdf**

**Lysosomal accumulation of polystyrene nanoparticles.**

Confocal microscopy analyses of polystyrene nanoparticles (green) and LAMP-2 (blue) in fibroblasts treated with polystyrene nanoparticles (50nm; 25 μg/mL), evaluated every 24 hr for up to 72 hr by detecting the fluorescence intensity of fluorescently labeled polystyrene nanoparticles and binding of anti-LAMP-2 antibody, respectively. Colocalization of polystyrene nanoparticles (green) and LAMP-2 (blue) is shown in merged images. UT, untreated. The scale bar is 20 μm.
