# Supplementary material for: The autophagic response to polystyrene nanoparticles is mediated by transcription factor EB and depends on surface charge
Source: J Nanobiotechnology. 2015 Nov 23;13:87. doi: 10.1186/s12951-015-0149-6 (PMC4657241; doi:10.1186/s12951-015-0149-6)
Supplement: Supplementary file 2 — 10.1186/s12951-015-0149-6 Polystyrene nanoparticle purchasing information. [file 12951_2015_149_MOESM2_ESM.pdf]

Song_AdditionalFile2.pdf

Polystyrene nanoparticle purchasing information

**Name**	**Functional Group**	**Size (nm)**	**Manufacturer**	**Cat. No.**
PS	--	50	Magsphere	PS050NM
PS*	--	50	Magsphere	PSF-050NM
PS-COOH	-COOH	50	Phosphorex	103
PS-COOH*	-COOH	50	Magsphere	CAF-050NM
PS-NH_2	-NH_2	50	Magsphere	AM050NM
PS-NH_2*	-NH_2	50	Magsphere	AMF-050NM

*Fluorescently (FITC) labeled nanoparticles
